# Case report: Genetic analysis of a novel intronic inversion variant in the *SPTB* gene associated with hereditary spherocytosis

**DOI:** 10.3389/fgene.2023.1309040

**Published:** 2023-12-04

**Authors:** Bixin Xi, Siying Liu, Yongbing Zhu, Dedong Zhang, Yu Zhang, Aiguo Liu

**Affiliations:** ^1^ Department of Pediatrics, Tongji Hospital, Tongji Medical College, Huazhong University of Science and Technology, Wuhan, China; ^2^ Department of Gynaecology, Wuhan Children’s Hospital (Wuhan Maternal and Child Healthcare Hospital), Tongji Medical College, Huazhong University of Science and Technology, Wuhan, China

**Keywords:** hereditary spherocytosis, *SPTB* gene, next-generation sequencing, intronic inversion variant, genetic analysis

## Abstract

**Background:** Hereditary spherocytosis (HS) is a congenital haemolytic anaemia attributed to dysregulation or abnormal quantities of erythrocyte membrane proteins. Currently, the most common erythrocytic gene, spectrin β (*SPTB*), variants are located in exons and give rise to mRNA defects. However, the genetic characteristics and pathogenic mechanisms of *SPTB* intronic variants are not completely understood. This study aimed to analyse a rare intronic inversion variant in the *SPTB* gene associated with HS, and explore the impact of the variant on *SPTB* mRNA splicing.

**Method:** The clinical manifestations of the patient were summarised and analysed for spherocytosis phenotype diagnosis. The pathogenic variant was identified in the proband using targeted next-generation and Sanger sequencing. RNA sequencing was performed to analyse whether *SPTB* gene splicing and expression were affected.

**Results:** Targeted next-generation sequencing identified a novel disease-associated intronic inversion variant of the *SPTB* gene in the proband. The inversion variant was located between intron 19 and 20, and contained the entire exon 20 and partial sequences of adjacent introns. Sanger sequencing confirmed that the intronic inversion variant only appeared in the genome of the proband, not in his parents. RNA sequencing revealed that the variant could result in the skipping of exon 20 and reduced expression of *SPTB* mRNA.

**Conclusion:** This study identifies a rare intronic inversion variant in the *SPTB* gene associated with hereditary spherocytosis. The pathogenic variant can lead to exon 20 skipping and decreased *SPTB* gene expression. This finding has not been previously reported in any literature. This study can expand the intronic variant spectrum of the *SPTB* gene, deepen our understanding of HS pathogenesis, and contribute to the genetic diagnosis and clinical management of patients.

## 1 Introduction

Hereditary spherocytosis is the most common congenital red cell membrane disorder resulting from pathogenic variants in five genes encoding erythrocyte membrane-anchoring and cytoskeleton proteins (Da Costa et al., 2013; Andolfo et al., 2016; Ji et al., 2019). The annual diagnosis of males and females with HS in China is approximately 1.27/100,000 and 1.49/100,000, respectively (Wang et al., 2015). However, the prevalence is likely underestimated given that spherocytosis is sometimes asymptomatic or has mild symptoms (Da Costa et al., 2013; Nieminen et al., 2021; Wu et al., 2021). Hereditary spherocytosis also refers to a group of heterogeneous non-immune anaemia showing a broad spectrum of severity in clinical manifestations that may easily result in misdiagnosis or missed diagnosis in some patients (Wu et al., 2021). Common symptoms of congenital spherocytosis include haemolytic anaemia, jaundice, and splenomegaly. Iron overload and cholelithiasis are the common symptoms of transfusion-dependent forms of congenital spherocytosis (Nieminen et al., 2021).

Pathogenic variants in five genes encoding ankyrin1 (*ANK1*), spectrin β (*SPTB*), spectrin alpha 1 (*SPTA1*), solute carrier family 4 member 1 (*SLC4A1*), and erythrocyte membrane protein band 4.2 (*EPB42*) are associated with erythrocyte membrane defects in patients with HS (He et al., 2018). The most common causative genes in Chinese patients with HS are *ANK1* (46%) and *SPTB* (42%), followed by *SLC4A1* (11%), whereas *SPTA1* and *EPB42* variants are less frequently reported (Wang D. et al., 2021). Autosomal dominant (AD) inheritance accounts for 75% of HS cases, mainly through *ANK1*, *SPTB* and *SLC4A1* gene variants, whereas autosomal recessive (AR) inheritance is described in approximately 25% of cases, frequently involving *SPTA1* and *EPB42* gene variants (Wang et al., 2020; Wang D. et al., 2021). β-spectrin (encoded by *SPTB*) plays a key role in the formation and mechanical stability of the erythrocyte membrane (He et al., 2018; Wang et al., 2020; Wang D. et al., 2021; Li et al., 2022). A total of 206 *SPTB* variants (mainly localised on exons) are reported in the Human Gene Mutation Database (HGMD, http://www.hgmd.cf.ac.uk/ac/index.php, accessed 26 March 2021). The common alterations of *SPTB* that give rise to mRNA defects and truncated β-spectrin include nonsense, frameshift, and splice site (Maciag et al., 2009).

*SPTB* gene deficiency is usually inherited in an AD manner (An and Mohandas, 2008). *De novo SPTB* variants are relatively infrequent in HS, and family investigations provide only limited clinical evidence (Wang et al., 2018; Wang J. F. et al., 2021). Two *de novo* point variants in the *SPTB* gene, causing aberrant mRNA splicing and exon skipping, have been identified in HS (Hassoun et al., 1996; Perrotta et al., 2009). One is localized on intron 17 and leads to truncated β-spectrin Winston-Salem. The other is localized on intron 16 and results in aberrant β-spectrin Bari. However, there is a paucity of information on the genetic characteristics and pathogenic mechanisms of *SPTB* intronic inversion variants.

This study aimed to analyse a rare intronic inversion variant in the *SPTB* gene associated with HS, and explore the impact of the variant on *SPTB* mRNA splicing. This study expands the *SPTB* intronic variant spectrum, deepens our understanding of the pathogenesis of HS, and provides a new perspective for the genetic diagnosis and clinical management.

## 2 Materials and methods

### 2.1 Patient and ethical approval

A patient with anaemia was recruited from Tongji Hospital, Wuhan, China. Detailed information, including family medical history, clinical manifestations, progression of HS, and the results of laboratory examinations, was collected. Blood samples were obtained and prior written approval of the subject and his parents were performed. All procedures were performed in accordance with the Declaration of Helsinki, and approved by the Ethics Committee of Tongji Hospital, Tongji Medical College, Huazhong University of Science and Technology.

### 2.2 Targeted next-generation sequencing (NGS)

Genomic DNA (1–3 μg) was extracted from peripheral blood samples of the proband and his parents using the QIAamp DNA Mini Kit (Qiagen, Shanghai, China). Each genomic DNA sample was segmented into fragments with an average size of 150 bp using an S220 Focused-ultrasonicator (Covaris, Woburn, MA, United States). Then, the DNA Sample Prep Reagent Set (MyGenostics, Beijing, China) was used to prepare paired-end sequencing libraries. Disease-causing gene variants were identified by customising an NGS panel targeting all exons and adjacent introns of HS-associated genes (*ANK1*, *SPTB*, *SPTA1*, *SLC4A1*, and *EPB42*), and a capture strategy was performed using the enrichment kit (MyGenostics Inc.) (Wu et al., 2011). Enriched libraries were sequenced using an Illumina HiSeq X 10 sequencer (Illumina, San Diego, CA, United States) (Song et al., 2019).

### 2.3 Bioinformatic analysis

Sequencing was followed by quality control to filter adapter sequences and low-quality reads (<80 bp). The filtered sequences were aligned to the human reference genome (hg19) using Burrows–Wheeler Aligner (BWA, http://bio-bwa.sourceforge.net/) (Li and Durbin, 2010). The single nucleotide variants (SNVs) and insertions/deletions (indels) were detected by Genome Analysis Toolkit (GATK, https://software.broadinstitute.org/gatk/) (McKenna et al., 2010). All the SNVs and indels were annotated with Annotate Variation (ANNOVAR, http://annovar.openbioinformatics.org/en/latest/), filtered using GATK Variant Filtration, and estimated via multiple databases (1000 Genomes Project, dbSNP, Exome Aggregation Consortium, Exome Variant Server, and HGMD). Functional prediction software was applied to predict the effect of the intronic inversion variant (Wu et al., 2015). Pathogenic variants were assessed according to the protocols issued by the American College of Medical Genetics and Genomics practice guidelines, in combination with clinical manifestations of the proband, family investigation, and screening tests for HS.

### 2.4 Sanger sequencing

Sanger sequencing was performed to verify the filtered variants. Specific primers for PCR were designed using Primer-BLAST (https://www.ncbi.nlm.nih.gov/tools/primer-blast/). The coding exons and adjacent introns of variant genes were amplified with Ex Taq DNA polymerase (Takara Bio, Dalian, China). Purified PCR products were sequenced on an ABI 3130 Genetic Analyzer (Applied Biosystems, CA), then analysed using the Mutation Surveyor (Softgenetics, PA). The same procedures were conducted in all family members to confirm the pathogenic variants.

### 2.5 RNA sequencing

Total RNA was extracted from the peripheral blood samples using TRIzol reagent (Invitrogen; Carlsbad, CA). Nanodrop (Thermo Fisher Scientific, Waltham, MA, United States) analysis and gel electrophoresis were performed to examine the RNA quality. The RNA library was constructed according to the manufacturer’s protocol (Illumina). Subsequently, RNA sequencing was performed to detect aberrant RNA expression events and potential changes in the splicing process. OUTRIDER (Outlier in RNA-Seq Finder, http://bioconductor.org/packages/OUTRIDER/) was used to identify aberrantly expressed genes in RNA sequencing data.

## 3 Results

### 3.1 Clinical features

The patient was a 7.5-year-old boy delivered full-term by caesarean section without asphyxia, weighing 3.6 kg, with a head circumference and body length of 34.7 cm and 51.3 cm, respectively. At birth, the local hospital detected a reduced haemoglobin level of 94 g/L (normal range [NR], 145–190 g/L) and progressive decline with 92 g/L and 79 g/L on days 3 and 6 after birth, respectively. Haemoglobin levels increased to 133 g/L after the infusion of red blood cells (RBCs). Haemoglobin levels were maintained at 85 g/L after discharge. At 4 months, the patient was readmitted to the hospital for “4 months of anaemia and 1 month of lagging growth”. Haematologic assessment revealed haemoglobin levels at 78.0 g/L (NR, 120–160 g/L), white blood cell count at 7.15 × 10^9^/L (NR, 3.5–9.5 × 10^9^/L), RBC count at 3.29 × 10^12^/L (NR, 4.0–5.5 × 10^12^/L), mean corpuscular haemoglobin concentration at 362 g/L (NR, 320–360 g/L), and reticulocytes at 4.95% (NR, 0.5%–1.5%). The indices of iron metabolism showed soluble transferrin receptors at a high level of 8.11 mg/L (NR, 2.2–5.0 mg/L), while other parameters were normal. The direct anti-human globulin test (Coombs test) found no abnormality. The patient had an ABO blood group A, Rh (D)-positive. The erythrocyte osmotic brittleness was elevated. Abdominal ultrasound showed an enlarged spleen. Total serum bilirubin (93.9 μmol/L; NR, <26.0 μmol/L) and indirect bilirubin (86.3 μmol/L; NR, <16.8 μmol/L) levels were elevated. The parents of the patient were not consanguineous and had no history of haematological disease. Their routine blood and serum biochemical tests were normal.

**FIGURE 1:**
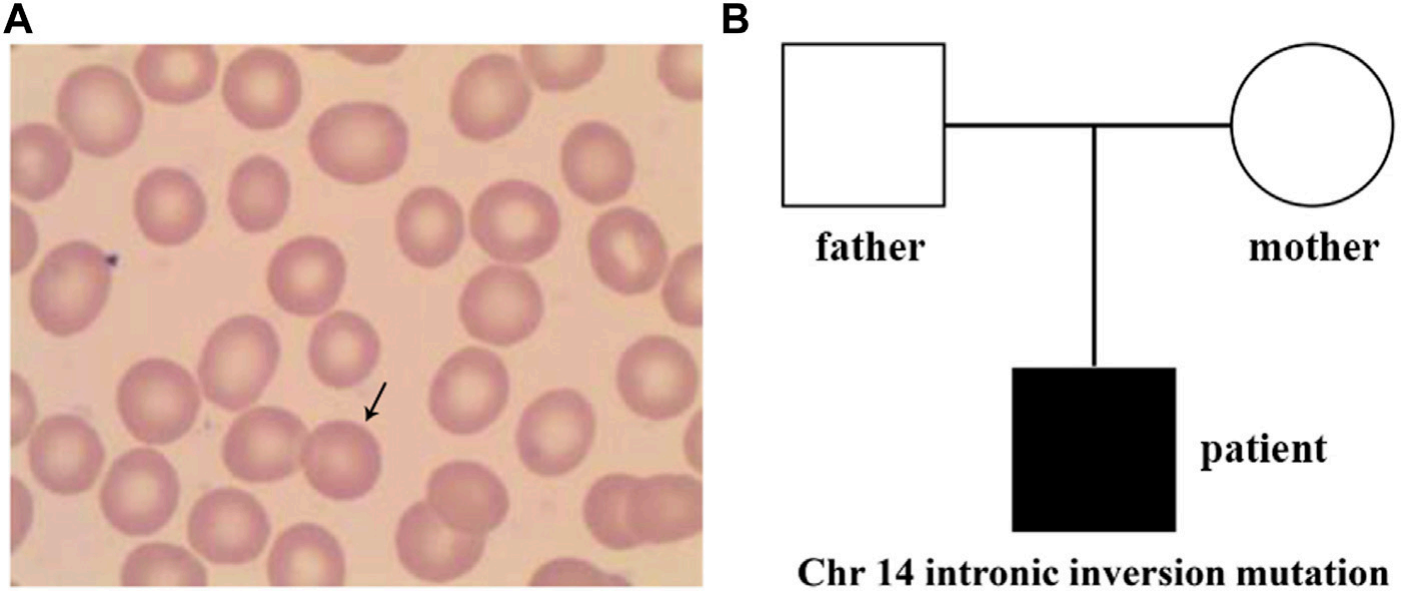
Peripheral blood smear and family tree of the patient. **(A)** Spherical-shaped erythrocytes were found in the peripheral blood film. **(B)**, Family tree of a Chinese family with the patient exhibiting hereditary spherocytosis. Square and circle denote male and female, respectively.

At the age of 7.5 years, clinical symptoms and abnormal laboratory results still existed, but jaundice and splenomegaly were more serious. The patient was readmitted to our hospital. Blood tests revealed a haemoglobin level of 89 g/L, with 3.01 × 10^12^/L RBCs and 10.69% reticulocytes. Serum biochemical tests revealed that the concentrations of total bilirubin (125.1 μmol/L) and indirect bilirubin (111.1 μmol/L) were higher than previous standards. Abdominal ultrasound showed aggravated splenomegaly. Bone marrow smear analysis showed obvious erythroid hyperplasia (39.2%), with a preponderance of middle and late erythroblasts. RBCs showed a disparity in size, and spherical-shaped erythrocytes (7%) were found in the peripheral blood film (Figure 1A). Glucose-6-phosphate dehydrogenase activity was normal. The *α*- and β-thalassemia genetic variant screen and haemoglobin electrophoresis were negative. The haematologic examinations are summarised in Table 1.

**TABLE 1:** Results of haematologic examinations of the patient.

Haematologic examinations	4 months	7.5 years	Normal range
Red blood cells (10^9^/L)	3.29	3.01	4.0–5.5
Haemoglobin (g/L)	89	89	120.0–160.0
Platelet (10^9^/L)	271	223	150.0–450.0
Haematocrit (%)	24.6	25.5	40.0–50.0
MCV (fL)	74.8	84.7	80.0–100.0
MCH (pg)	27.1	29.6	27.0–34.0
MCHC (g/L)	362	349	320.0–360.0
Reticulocyte percentage (%)	4.95	10.69	0.5–1.5
Reticulocyte (10^12^/L)	0.163	0.322	0.025–0.075
Total bilirubin (µmol/L)	93.9	125.1	<26.0
Indirect bilirubin (µmol/L)	86.3	111.1	<16.8
LDH (U/L)	277	303	120.0–300.0
FHb (mg/L)	<25.0	<25.0	<40.0
Ferritin (µg/L)	121.9	438.9	30.0–400.0
G6PD (U/L)	ND	2036	>1300
Coombs test	negative	negative	negative
Erythrocyte osmotic fragility test	ND	positive	negative
Haemoglobin electrophoresis	ND	negative	negative

MCV, mean corpuscular volume; ND, not done; MCH, mean corpuscular haemoglobin; MCHC, mean corpuscular haemoglobin concentration; LDH, lactate dehydrogenase; FHb, free haemoglobin; G6PD, glucose-6-phosphate dehydrogenase; Coombs test, direct anti-human globulin test.

### 3.2 Genetic and phenotype analysis

#### 3.2.1 Next-generation sequencing output and coverage

Next-generation sequencing was performed with a gene panel targeting all coding exons and adjacent introns of genes associated with haematological system disease, including *ANK1*, *SPTA1*, *EPB42*, *SLC4A1*, and *SPTB*. Sequencing of the 99.80% targeted amplicons of the proband were covered at least once, with 99.56%, 99.22%, and 98.63% amplicons covered at least 4, 10, and 20 times, respectively. The mean read depth was 531-fold.

#### 3.2.2 Mutation detection

**FIGURE 2:**
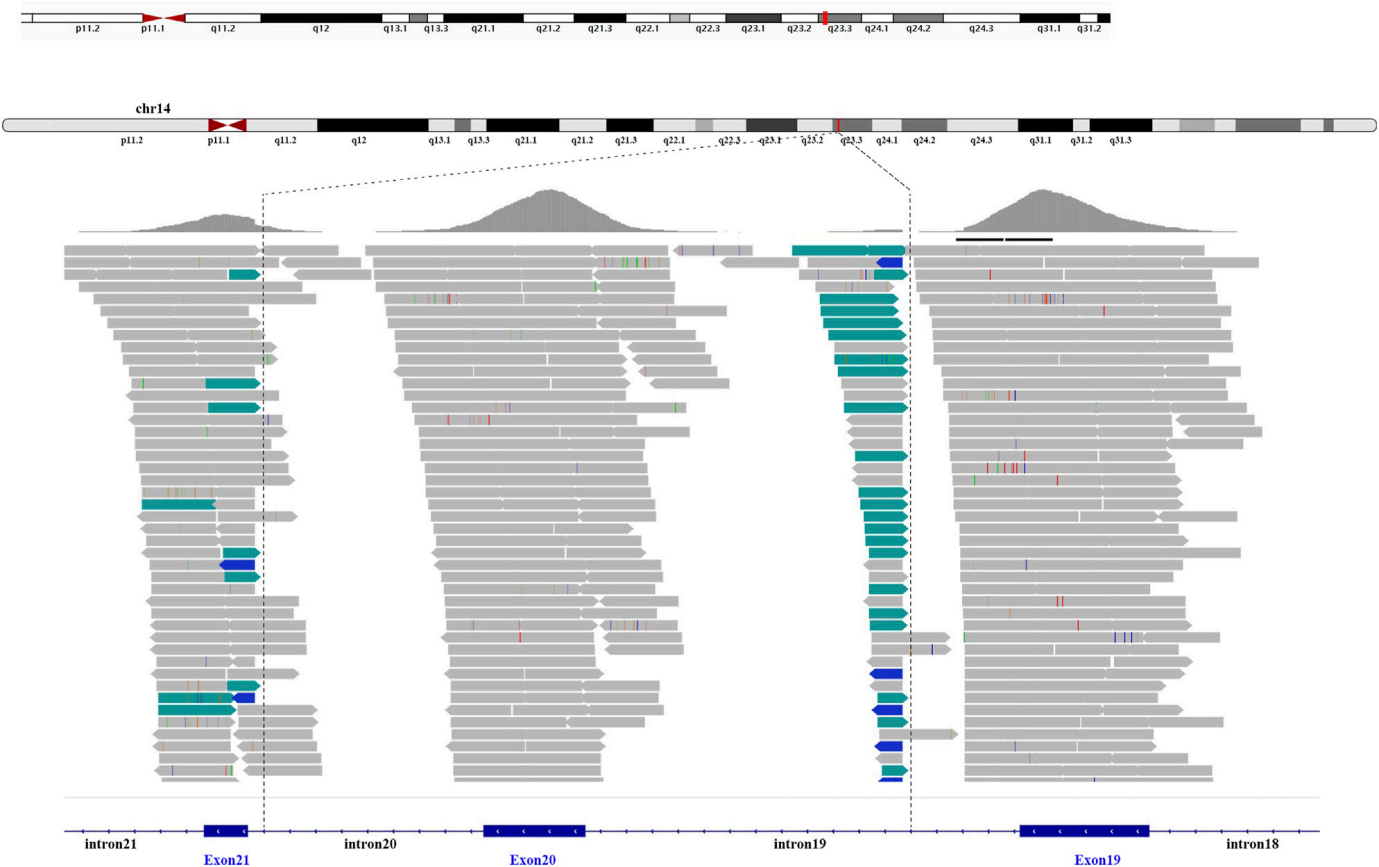
Next-generation sequencing. The intronic inversion mutation located between intron 19 and 20 of the *SPTB* gene was visualized by the integrative genomic viewer. The discordant reads are labelled green and blue.

**FIGURE 3:**
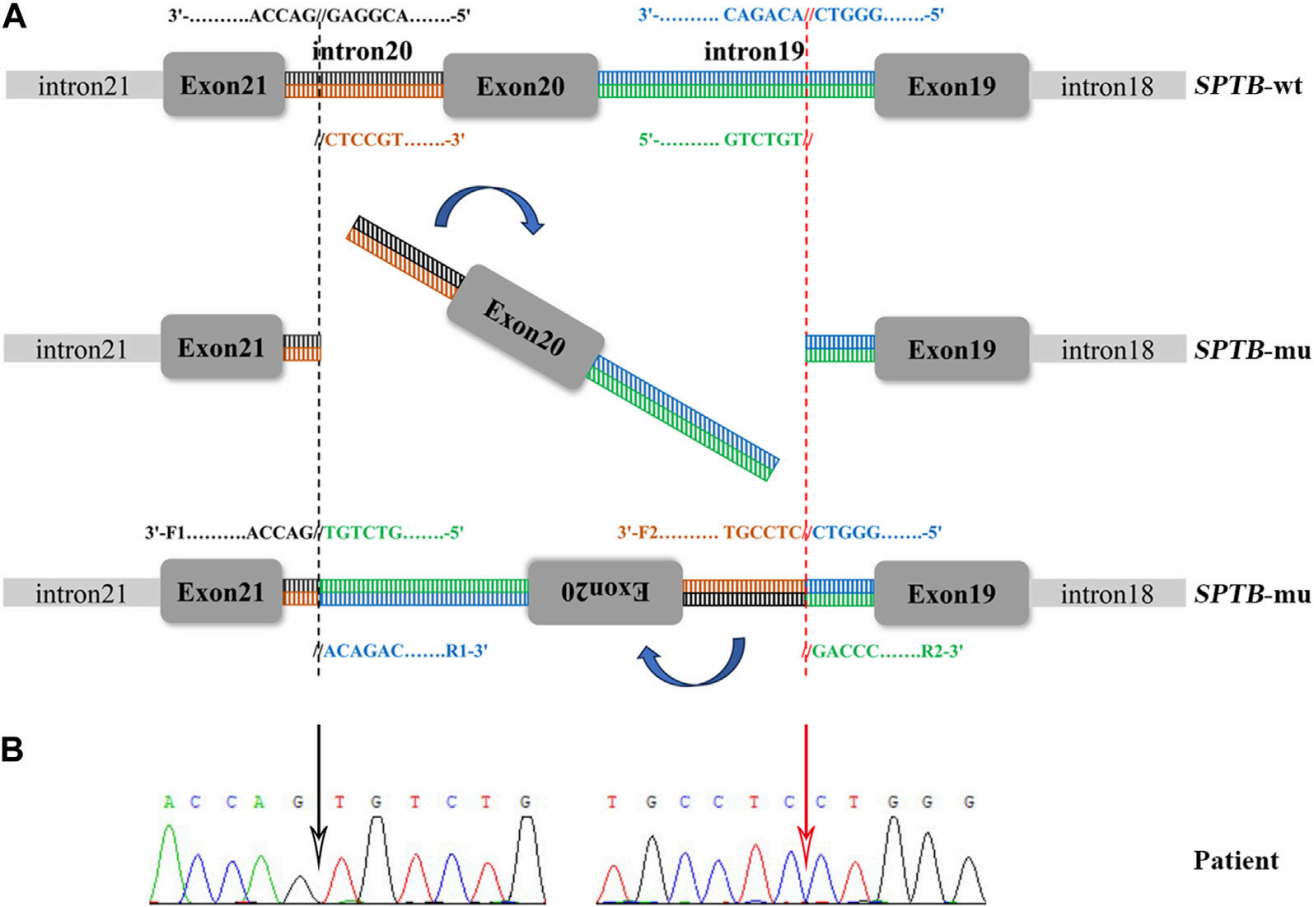
Sanger sequencing validation and a schematic diagram of the intronic inversion mutation in *SPTB*. **(A)** Schematic diagram of the *SPTB* gene. F1, forward primer 1; R1, reverse primer 1; F2, forward primer 2; R2, reverse primer 2. **(B)** The intronic inversion variant carried by the proband was confirmed using Sanger sequencing. The black and red arrow indicates the 5′-gene breakpoint of intron 19 and 3′-gene breakpoint of intron 20 in the *SPTB* gene, respectively.

A total of 36 variants in the patient were identified using NGS (Supplementary Table S1), filtered, and annotated to detect pathogenic variants. A novel intronic inversion in the *SPTB* gene was identified, which was associated with the patient’s phenotype. The inversion variant resided between the 5′-gene breakpoint of intron 19 and 3′-gene breakpoint of intron 20 in the *SPTB* gene (Figure 2; Figure 3A) that could disrupt the canonical splice site. This inversion variant contained entire exon 20 and partial sequences of adjacent introns. The inversion was represented with a schematic diagram (Figure 3A).

#### 3.2.3 Sanger sequencing validation and RNA sequencing analysis

**FIGURE 4:**
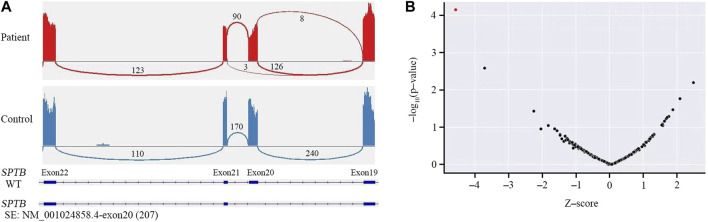
RNA sequencing analysis. **(A)** RNA sequencing showed skipped exon 20 in the *SPTB* mRNA of the patient, but not the control. **(B)** Gene-level significance (−log_10_(P), *y*-axis) versus Z-score, with *SPTB* labelled as the expression outlier (red dot) of sample from the proband.

Sanger sequencing was performed on the cDNA samples from all family members to validate the intronic inversion variant in the *SPTB* gene using the following primers: 3′-gene breakpoint of intron 20 in *SPTB*: forward primer 1, 5′- AGG​ACT​CAC​CTG​GTT​CTT​CTT​CAT​G-3′ and reverse primer 1, 3′- TGA​TGC​AGA​AAC​AGA​AAA​CC-5'; 5′-gene breakpoint of intron 19 in *SPTB*: forward primer 2, 3′-GGA​GCA​AGT​TAT​TTG​GAA​CCT​CAG-5′ and reverse primer 2, 3′- ACA​AAG​GAG​AAG​ACC​CAG​CAC​C-5' (Figure 3A). The PCR product only presented in the proband, not in his parents (Figure 1B; Figure 3B). This confirmed that the intronic inversion was a *de novo* mutation. RNA sequencing analysis found that the intronic inversion variant was associated with exon 20 skipping in the *SPTB* mRNA (Figure 4A), and revealed one underexpression outlier, which was the *SPTB* gene (Figure 4B).

#### 3.2.4 Functional prediction and phenotype analysis

The *de novo* intronic inversion in *SPTB* was not found in the 1000G, ExAC, EVS, or HGMD databases. Functional prediction software was applied to predict the effect of the intronic inversion variant. REVEL, SIFT, and MutationTaster predicted the variant to be “disease causing”, while PolyPhen predicted the variant to be “probably damaging.” Additionally, haemolysis, splenomegaly, jaundice, spherically-shaped erythrocytes of peripheral blood film, and increased erythrocyte osmotic brittleness were observed in the patient, which were consistent with the *SPTB* genetic variant.

## 4 Discussion

A precise diagnosis of HS is challenging owing to the heterogeneity of clinical manifestations and non-ideal sensitivity or specificity of some diagnostic tests. Misdiagnosis or missed diagnosis of HS still existed in some patients in recent years (Ma et al., 2018; Zhu et al., 2020). The conventional HS diagnosis scheme is mainly based on family history, typical clinical manifestations, and relevant laboratory test results. However, the clinical manifestations of HS in children show obvious heterogeneity. Most patients suffer from well-compensated haemolytic anaemia, while only a minority have severe anaemia requiring blood transfusion (Wu et al., 2021). Routine blood tests, such as low haemoglobin, normal or elevated reticulocytes, normal or increased mean corpuscular haemoglobin concentration, increased number of spherocytes, and elevated indirect bilirubin in the serum are not sufficiently sensitive or specific (Wu et al., 2021). The flow cytometric osmotic fragility test (FOFT) and eosin-5′-maleimide dye-binding test (EMA) have been shown to be superior to conventional osmotic fragility tests for the diagnosis of HS (Arora et al., 2018). In our case, the proband had a negative family history and lack of typical microspherocytes (<10.0%); therefore, he was initially misdiagnosed with autoimmune haemolytic anaemia. Next-generation sequencing facilitated fast and accurate detection of the variant sites of disease-causing genes. This is the first study precisely identifying pathogenic intronic inversion in the *SPTB* gene using NGS, confirming its clear advantage over conventional inspection methods in diagnosis. Thus, the new HS diagnostic protocol (clinical manifestations, routine laboratory tests, a combination of FOFT and EMA, family investigation, and genetic testing) could improve the clinical efficiency and accuracy of HS diagnosis.

The *SPTB* gene (OMIM accession number 182870) is located on chromosome 14q23.3, which is over 100 kbp long, and contains 35 exons encoding 2,328 amino acids that make up an actin-binding region, seventeen spectral repeats, and a C-terminus (Ipsaro et al., 2009; Wang D. et al., 2021). In normal human erythrocytes, *a*-spectrin (encoded by *SPTA1*) has a synthesis rate that is three to four times faster than β-spectrin (encoded by *SPTB*). Thus, β-spectrin synthesis is a rate-limiting step for the formation of the α2β2-tetramerisation domain (Perrotta et al., 2008; He et al., 2018). β-spectrin plays a crucial role in maintaining deformability and stability of the erythrocyte membrane. *SPTB* variants can lead to failures in erythrocyte membrane skeleton formation, decreases in the reversible deformation ability of RBCs, and the destruction of erythrocytes as they traverse the microvasculature and splenic tissues (Tole et al., 2020). *ANK1* variants are only identified in HS, while *SPTB* variants are found in other diseases, such as hereditary elliptocytosis or hereditary pyropoikilocytosis (He et al., 2018). *SPTB* variants can be distributed throughout the gene; most of the variant sites are located outside the tetramerisation domain (encoded by exons 1–7), followed by spectrin repeats (encoded by exons 8–30) (Park et al., 2016; Wang et al., 2020). A retrospective study of 66 cases of HS showed that all *SPTB* variants were distributed outside the tetramerisation domain, except four variants associated with the hereditary elliptocytosis phenotype and two variants located in the spectrin repeats domain (Wang D. et al., 2021). These variants in the *SPTB* gene were mainly nonsense (32/66) and frameshift (15/66), which could lead to the deficiency of *SPTB* haploids and normal β-spectrin. This study also revealed that most *SPTB* variants were localised on exons 13, 15, and 18–30. Another study regarding 38 unrelated HS patients found 17 *SPTB* variants that mainly include nonsense variants (8/17), followed by splicing variants (3/17), duplications (2/17), missense variants (2/17), indel (1/17), and small deletion (1/17) (Wang et al., 2018). In addition, most of these *SPTB* variants were located on exons (15/17) and are considered as null variants that may result in *SPTB* haploinsufficiency and β-spectrin deficiency.

To date, most of the gene defects in *SPTB* (splicing, nonsense variants, and frameshift variants) usually result in exon skipping, mRNA transcript instability, or synthesis of truncated β-spectrin proteins (Hassoun et al., 1997; Perrotta et al., 2009). In a large family with 22 HS patients, researchers found that the intronic variant (*SPTB* NP_001020029.1: p.Ile294Serfs*35) led to exon 8 skipping, aberrant mRNA splicing, and synthesis of truncated proteins lossing seventeen spectral repeat domains (Nieminen et al., 2021). Another study on intronic *SPTB* variants showed that the β-spectrin deficiency were also associated with the instability of aberrantly spliced mRNA and the haploinsufficiency of *SPTB* (Maciag et al., 2009). Our study identified a novel intronic inversion of the *SPTB* gene in the HS proband. The inversion variant was located between intron 19 and 20, and contained the entirety of exon 20 and partial sequences of adjacent introns. This rare pathogenic inversion was not identified in any other databases. The intronic inversion variant could lead to the skipping of exon 20, a decrease of *SPTB* gene expression, and the associated spherocytosis phenotype.

However, the specific mechanism through which the pathogenic inversion localized on introns leads to exon skipping, aberrant mRNA splicing and β-spectrin deficiency remains unclear. It is suggested that the intron splice order and speed were crucial factors in the skipping of multiple exons and synthesis of truncated proteins (Takahara et al., 2002). The half-life and stability of truncated β-spectrin protein is worse than that of the wild-type, which contributes to increased proteolytic degradation of the truncated-type (Hassoun et al., 1996; Bogusławska et al., 2017). In addition, it was previously reported that the aberrant *SPTB* mRNA could not be detected in some HS cases, which may be due to transcript instability (Bassères et al., 2002; Bogusławska et al., 2017). Insufficient sample size and transcript instability were challenges for our study. Further studies on HS pathogenesis are urgently needed.

This study can also provide a theoretical basis for child healthcare, genetic counselling, prenatal diagnosis, and molecular targeted therapy into HS. A structured follow-up was recommended for the patient consisting of blood tests and abdominal ultrasound evaluations in the haematology outpatient clinic, together with visits by the spleen surgeon and child healthcare specialists every 3 months. The patient’s clinical condition has remained stable to date, and all family members are satisfied with the outcomes and agreed to regular follow-ups.

## 5 Conclusion

This study identified a rare intronic inversion variant localised between intron 19 and 20 of the *SPTB* gene that associated with the spherocytosis phenotype. It is the first study accurately detecting a pathogenic intronic inversion variant via NGS in China. This improves the clinical efficiency and accuracy of HS diagnosis, and confirms its clear advantage over conventional inspection methods. Moreover, RNA sequencing analysis revealed that the variant could lead to the skipping of exon 20 and decreasing of *SPTB* gene expression, which provided a new perspective for the pathogenesis of hereditary spherocytosis. These findings can ultimately lead to more informed healthcare and genetic counselling outcomes and better prognosis for patients with HS.

## Data Availability

The datasets for this article are not publicly available due to concerns regarding participant/patient anonymity. Requests to access the datasets should be directed to the corresponding authors.
