# Supplementary material for: Case report: Genetic analysis of a novel intronic inversion variant in the SPTB gene associated with hereditary spherocytosis
Source: Front Genet. 2023 Dec 4;14:1309040. doi: 10.3389/fgene.2023.1309040 (PMC10726134; doi:10.3389/fgene.2023.1309040)
Supplement: Supplementary file 1 [file Table1.pdf]

Supplementary Table 1 | Details of 36 variants detected in the patient using next generation sequencing.

Gene Symbol	ID	Ref Transcript	Exon/intron	Nucleotide Changes	Amino Acid Changes	Inhert	Gene Type
NUP214	chr9:134006159	NM_005085	exon5	c.599G>A	p.W200X	AR	het
HK1	chr10:71146158	NM_000188	exon13	c.1919C>T	p.A640V	AR, AD	het
FANCG	chr9:35078604	NM_004629	exon3	c.305G>A	p.R102K	AR	het
KMT2C	chr7:151935799	NM_170606	exon15	c.2645T>C	p.I882T	AD	het
KMT2C	chr7:151935871	NM_170606	exon15	c.2573G>T	p.W858L	AD	het
DIAPH1	chr5:140907267-140907269	NM_001079812	exon23	c.3122-5_3122-3delTTT	splicing	AR, AD	het
CDH23	chr10:73501595	NM_022124	exon36	c.4762C>T	p.R1588W	AR, AD	het
KMT2C	chr7:151935866	NM_170606	exon15	c.2578C>T	p.P860S	AD	het
MPO	chr17:56350912	NM_000250	exon9	c.1484C>T	p.T495I	AR, AD	het
VPS13A	chr9:79922972	NM_033305	exon35	c.4072G>A	p.A1358T	AR	het
ERBB3	chr12:56489543	NM_001982	exon17	c.2008C>T	p.R670W	AR, AD	het
IL6ST	chr5:55243420-55243420	NM_001364279	exon13	c.845-4_845-3insTT	splicing	AR	het
BCR	chr22:23630368	NM_021574	exon12	c.2602+9C>T	splicing	-	het
MYO5A	chr15:52645860	NM_001382349	exon28	c.3640-5C>T	splicing	AR	het
SPTB	chr14:65245973-65248767	NM_001024858	intron19, intron20	-	splicing	de novo	het
RTEL1	chr20:62324315	NM_032957	exon29	c.2882C>G	p.P961R	AD, AR	het
HPS6	chr10:103826163	NM_024747	exon1	c.932C>T	p.P311L	AR	het
USH2A	chr1:216073426	NM_206933	exon40	c.7585G>A	p.A2529T	AR	het
SLX4	chr16:3633191	NM_032444	exon14	c.5060C>G	p.P1687R	AR	het
CARMIL2	chr16:67685613	NM_001013838	exon25	c.2453C>T	p.T818I	AR	het
ERCC4	chr16:14015921	NM_005236	exon2	c.241G>T	p.V81F	AR	het
GLB1	chr3:33118581	NM_001317040	exon2	c.219+5G>A	splicing	AR	het
GPI	chr19:34868498	NM_001289789	exon6	c.603+7A>G	splicing	AR	het
PHKB	chr16:47621684	NM_001363837	exon9	c.870+10T>A	splicing	AR	het
DKC1	chrX:154005089-154005091	NM_001363	exon15	c.1492_1494delAAG	p.498delK	XLR	het
UGT1A1	chr2:234669144	NM_000463	exon1	c.211G>A	p.G71R	AR	het
GP1BA	chr17:4837660	NM_000173	exon2	c.1761A>C	p.Q587H	AD, AR	het
VWF	chr12:6127535	NM_000552	exon28	c.5049A>C	p.A1683A	AD, AR	het
USH2A	chr1:216270422	NM_206933	exon22	c.4758+3A>G	splicing	AR	het
BCR	chr22:23654032	NM_021574	exon18	c.3190+9T>C	splicing	-	het
SLC2A2	chr3:170715865	NM_000340	exon11	c.1402C>G	p.L468V	AR, AD	het
WRN	chr8:30949398	NM_000553	exon16	c.1882C>G	p.L628V	AR	het
HMBS	chr11:118962230	NM_000190	exon9	c.606G>T	p.V202V	AD	het
NCF1	chr7:74193642	NM_000265	exon4	c.269G>A	p.R90H	AR	het
FECH	chr18:55226383	NM_000140	exon7	c.798C>G	p.P266P	AR	hom
HK1	chr10:71146158	NM_000188	exon13	c.1919C>T	p.A640V	AR, AD	het
